# A prostate cancer–specific super-enhancer at 1q42.3 regulates the *CAPN2* and *KRT19*

**DOI:** 10.1016/j.jbc.2026.113588

**Published:** 2026-09-23

**Authors:** Zhenhao Zhao, Yunlong Bai, Qin Wang, Yuting Zhao, Yuan Jiang, Hongxia Li, Zeyun Yang, Xuan Liu, Lei Zhang, Zhongfang Zhao, Yuyang Qian, Peng Li, Jiandang Shi, Wange Lu

**Affiliations:** 1 College of Life Sciences, State Key Laboratory of Medicinal Chemical Biology, Frontiers Science Center for Cell Responses, Nankai University, Tianjin, People's Republic of China; 2 Department of Head and Neck Oncology, Tianjin Medical University Cancer Institute & Hospital, National Clinical Research Center for Cancer, Tianjin's Clinical Research Center for Cancer, Key Laboratory of Basic and Translational Medicine on Head & Neck Cancer, Tianjin, Tianjin, People's Republic of China; 3 Department of Laboratory Medicine and Institute of Precise Medicine, The First Affiliated Hospital, Sun Yat-sen University, Guangzhou, Guangdong, People's Republic of China

**Keywords:** 1q42.2-1q43 locus, super-enhancer, NR125945-SE, long-range chromatin interactions, prostate cancer

## Abstract

Genetic susceptibility plays an essential role in prostate cancer (PCa) risk, while the functional mechanisms underlying several classic risk loci remain largely uncharacterized. Here, we identified a PCa specific super-enhancer within 1q42.3 locus, termed NR125945-SE, which resides within a previously reported PCa susceptibility region. Through comparative H3K27ac ChIP-seq analysis, we observed that NR125945-SE is highly active in PCa cells compared to normal controls, and this super-enhancer region is also epigenetically activated in human prostate cancer tissues. NR125945-SE contributes to PCa malignant progression. Mechanistically, integrative analysis combining RNA-seq and 4C-seq data revealed that NR125945-SE regulates the expression of *CAPN2* which encoding the calpain-2 protease, and *KRT19* which encoding keratin 19, through long-range chromatin interactions. Over expression of these candidate downstream targets partially rescued the transcriptional and phenotypic consequences of NR125945 deletion. Furthermore, we demonstrated that NR125945-SE activity is mediated by the cooperative binding of multiple transcription factors, including ETV5 and FOXK1, which physically occupy this super-enhancer. Consistently, elevated expression of *CAPN2* and *KRT19* was associated with poor biochemical recurrence–free survival and advanced castration-resistant prostate cancer in patient cohorts. In summary, our findings provide insights into a functional epigenetic element within a classic genetic risk locus, proposing a mechanism by which super-enhancer-mediated regulation promotes prostate cancer progression.

Prostate cancer (PCa) is the most prevalent malignancy in men and ranks as the second leading cause of cancer-related death in the United States (1). Accumulating evidence indicates that genetic factors account for a large proportion of PCa susceptibility, accounting for up to 42% of disease risk (2, 3). Genome-wide association studies (GWAS) and linkage analysis identified multiple PCa susceptibility loci, including 1q23–25, 1q42–43, 8p22–23 and 17p (4, 5, 6, 7). Several candidate risk genes have been characterized within the 1q23–25, 8p22–23 and 17p regions, such as *ELAC2*, *RNASEL* and *MSR1* (8, 9, 10). In contrast, although the 1q42.3 locus has long been identified in prostate cancer susceptibility, the previously reported candidate gene *PCTA-1* does not exhibit strong risk-associated features in prostate cancer (11). Therefore, the functional mechanisms underlying the 1q42.2-1q43 locus remain largely uncharacterized which may contribute to PCa progression.

Beyond protein-coding variation, gene expression in eukaryotic genomes is regulated by a complex set of epigenetic regulatory elements (12, 13, 14, 15, 16). Enhancers are critical cis-regulatory DNA elements that modulate chromatin activation states and typically marked by high level of H3K27ac. By orchestrating precise spatial and temporal patterns of gene expression, enhancers play essential roles in regulating the progression of diseases and determining cell identity (17, 18, 19, 20, 21). Super-enhancers are large clusters of enhancers with high transcriptional activity (22). The aberrant activation of super-enhancers (SEs) has emerged as a central driver of transcriptional dysregulation in cancer (23). In prostate cancer, SE landscapes are extensively rewired to drive malignant phenotypes and therapy resistance. For instance, AR binds to the *CHPT1*-associated SE locus and facilities androgen-independent *CHPT1* expression through recruiting BRD4 during enzalutamide resistance (24, 25). Consistently, perturbation of enhancer–promoter interactions underlie the regulation between *AR* and *MYC*, contributing to castration-resistant growth (26). Importantly, part of the susceptibility risk SNPs are enriched in these SE regions, suggesting that genetic risk variants may have an effect by disrupting or activating the activity of SE (27). While these findings underscore the critical role of SEs in PCa biology, the function of SEs located within specific genetic risk loci remains largely unexplored.

To address this gap, we profiled the enhancer landscape of PCa by integrating H3K27ac ChIP-seq data across normal and malignant prostate cell lines. This analysis identified a group of PCa-enriched SEs predominantly located in noncoding regions and displayed elevated H3K27ac signals across multiple PCa cell lines. Among them, the 1q42.3 locus NR125945-SE emerged as a candidate, which displayed strong chromatin accessibility and resides within a region that has been consistently associated with PCa susceptibility in prior genetic studies (7, 28, 29). Based on these insights, we hypothesized that the 1q42.2-1q43 susceptibility region may contain a tumor-specific super-enhancer that drives PCa progression through long-range regulation. Although PCa risk loci has been extensively studied, dysregulation of functional non-coding elements within these loci remains comparatively underexplored. Therefore, systematic characterization of the epigenetic landscape within these risk loci is essential for current understanding of prostate cancer.

In this study, we investigated the epigenetic landscape of the 1q42.2-1q43 region. Within this susceptibility region, we identified a novel tumor-specific super-enhancer (SE) through comparative analysis of H3K27ac ChIP-seq profiles which is highly co-localized with the non-coding transcript NR125945.1, hereafter referred to as NR125945-SE. NR125945-SE promotes cell proliferation and migration in PCa cell lines and is epigenetically activated in human prostate cancer tissues. Integrated analysis of RNA-seq and 4C-seq, combined with rescue experiments revealed that NR125945-SE regulates *CAPN2* and *KRT19* expression through long-range chromatin interactions, identifying these genes as candidate downstream targets. Furthermore, NR125945-SE is regulated by the cooperative binding of multiple transcription factors. Collectively, our findings provide insight into the functional epigenetic element within a classic genetic risk locus, proposing a mechanism by which super-enhancer–mediated regulation promotes prostate cancer progression.

## Results

### Identification of NR125945-SE as a tumor-specific super-enhancer at the 1q42.2-1q43 prostate cancer susceptibility locus

To investigate the role of SEs and the epigenetic regulation in PCa tumorigenesis, we conducted a comparative analysis of public H3K27ac chromatin immunoprecipitation followed by sequencing (ChIP-seq) data derived from four PCa cell lines (22Rv1, C4-2B, LNCaP, and VCaP) and two normal prostate epithelial cell lines (PrEC and RWPE1) (Fig. 1*A*). In 1q42.2-1q43 region, we analyzed the significantly enriched H3K27ac peaks in PCa cell lines. Among the upregulated peaks, we focused on the peaks overlapping with SE regions which indicated the genomic locus with extraordinary transcriptional activation potential and may function as a predominant regulatory hub (Fig. 1*B*) (30, 31). The peaks mapped to NR125945-SE region possess the highest score (Fig. 1*C*). Consistently, the ChIP-seq analysis showed that NR125945-SE region posed specifically and contiguously enriched H3K27ac signals in PCa cell lines and prostate patient tissues (Fig. 1*D* and S1*A*). The non-coding transcript NR125945.1 is consistently expressed at low levels across prostate cancer cell lines and clinical PRAD samples (Fig. S1, *B* and *C*), indicating limited transcriptional output from this locus. Accordingly, we focused on the regulatory function of the underlying super enhancer, hereafter referred to NR125945-SE. Furthermore, applying the ROSE algorithm classified this region as a super-enhancer across all four PCa cell lines (Fig. 1*E*). These findings strongly suggest that NR125945-SE may function as a critical regulatory element driving the malignant progression of prostate cancer.

**Figure 1:**
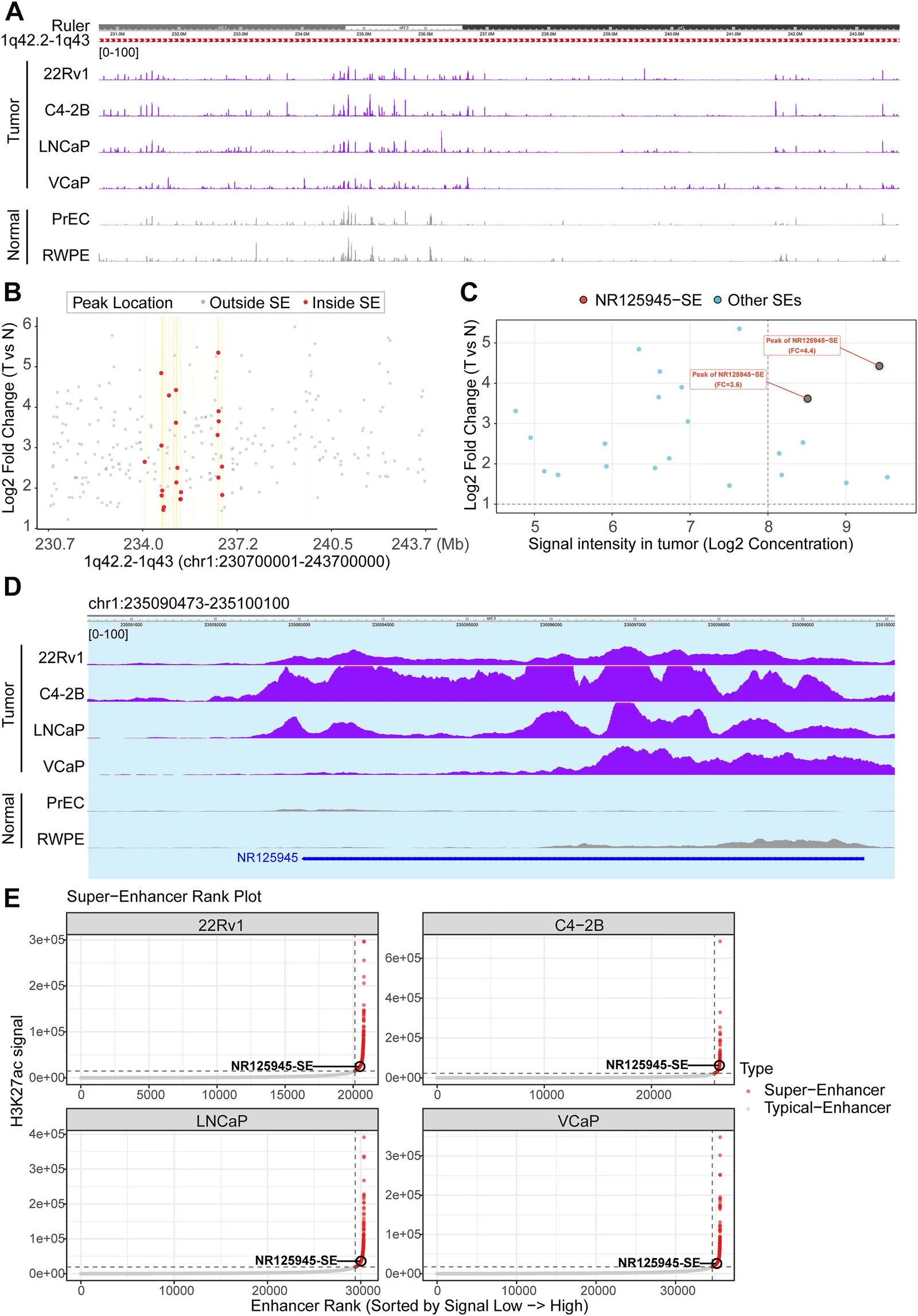
**Identification of NR125945-SE as a tumor-specific super-enhancer at the 1q42.2-1q43 prostate cancer susceptibility locus.***A*, comparative visualization of H3K27ac ChIP-seq enrichment profiles across two normal prostate epithelial cell lines (PrEC and RWPE1) and four prostate cancer (PCa) cell lines (22Rv1, C4-2B, LNCaP, and VCaP). *B*, significantly upregulated H3K27ac peaks in 1q42.2-1q43 region in PCa cell lines. *Red dots* represent peaks overlapped with SE regions. *Gray dots* represent peaks nonoverlapped with SE regions. *C*, ranking of upregulated H3K27ac peaks in 1q42.3-1q43 region. The top two peaks are located at NR125945-SE region. *D*, the tracks showed an aberrantly active chromatin domain spanning approximately 10 kb within the 1q42.2-1q43 region in PCa cell lines. *E*, rank ordering of super-enhancers (ROSE) analysis identifies the NR125945 associated region as a super-enhancer across all four PCa cell lines.

### NR125945-SE KO impairs malignant phenotypes in PCa

To investigate the functional role of NR125945-SE, we generated CRISPR/Cas9 mediated knockout (KO) to delete approximately 10 kb intergenic region that includes the entire active H3K27ac peaks in 22Rv1 cells (Fig. 2*A*). We successfully obtained three cell lines (KO-#1-3) with complete knockout of this super-enhancer, which were validated by genomic PCR (Fig. 2*B*).

**Figure 2:**
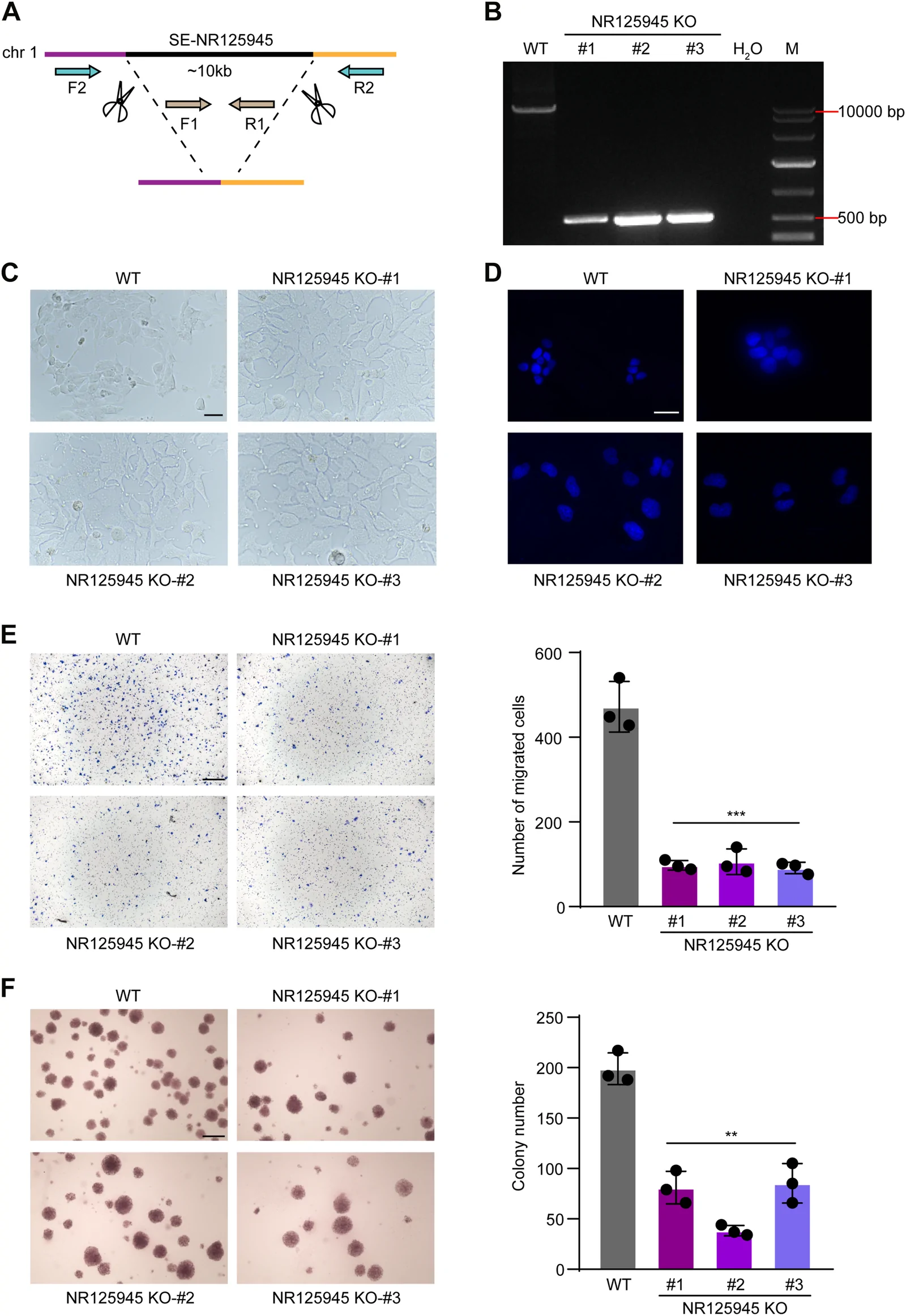
**CRISPR/Cas9-mediated deletion of the 1q42.3 super-enhancer impairs malignant phenotypes in prostate cancer cells.***A*, schematic illustration of the CRISPR/Cas9-mediated deletion strategy targeting the core region of the NR125945 super-enhancer (SE) and the location of validation primers. *B*, agarose gel electrophoresis confirming SE deletion using external primer pairs (F2+R2). M, DNA marker. *C*, representative bright-field images showing the morphological differences between WT 22Rv1 cells and three independent NR125945-SE KO clones. Scale bar, 20 μm. *D*, Hoechst staining comparing nuclear morphology between wild-type 22Rv1 cells and SE-KO clones. Scale bar, 20 μm. *E*, Transwell migration assay showing decreased migrated ability of NR125945-SE KO cells compared with WT 22Rv1. Migrated cells were stained with 0.1% crystal *violet*. Quantification (*right*) represents mean ± SD. ∗∗∗*p* < 0.001. Scale bar, 100 μm. *F*, soft agar colony formation assay showing markedly reduced clonogenic capacity of SE-KO cells relative to wild-type 22Rv1. Quantification (*right*) represents mean ± SD. ∗∗*p* < 0.01. Scale bar, 100 μm.

Functionally, NR125945-SE KO increased cell volume and reduced membrane definition relative to 22Rv1 wild-type (WT) controls (Fig. 2*C*). Meanwhile, Hoechst staining demonstrated that NR125945-SE KO cells exhibited significant changes in nuclear morphology compared with WT cells. Nuclear enlargement and loss of morphological regularity were observed upon NR125945-SE KO (Fig. 2*D*). These results suggested that knockout of the super-enhancer likely perturbs chromatin structure and distribution, which may consequently lead to aberrant gene expression and ultimately impair critical biological functions. Moreover, Transwell assay showed that the invasive migration was significantly reduced in KO compared with WT 22Rv1 cells (Fig. 2*E*). Relevant to colony formation, soft agar assay showed that KO cells exhibited significantly reduced colony formation capacity compared to WT 22Rv1 cells (Fig. 2*F*). Together, these results indicated that the NR125945-SE is indispensable for sustaining malignant phenotypes of prostate cancer cells.

### Loss of NR125945-SE induces global transcriptional alterations in 22Rv1

To further investigate the molecular consequences of NR125945-SE KO, we performed RNA-sequencing (RNA-seq) in WT and NR125945-SE KO cell lines. Compared to the WT, NR125945-SE KO exhibited 972 genes downregulated and 538 genes upregulated. KEGG pathway analysis indicated that the down-regulated genes based on NR125945-SE KO were significantly enriched in pathways such as neuroactive ligand–receptor interactions, cell adhesion molecules, and ECM-receptor interactions (Fig. 3*C*). Gene ontology (GO) analysis showed that the down-regulated genes were significantly enriched in biological processes such as signal transduction and cell adhesion (Fig. 3*D*). Gene set enrichment analysis (GSEA) also revealed that NR125945-SE KO selectively alters genes enriched in pathways governing epithelial–mesenchymal transition (EMT) and apical surface signaling (Fig. 3, *E* and *F*). These results demonstrated that NR125945-SE is a critical regulatory element involved in various important biological processes.

**Figure 3:**
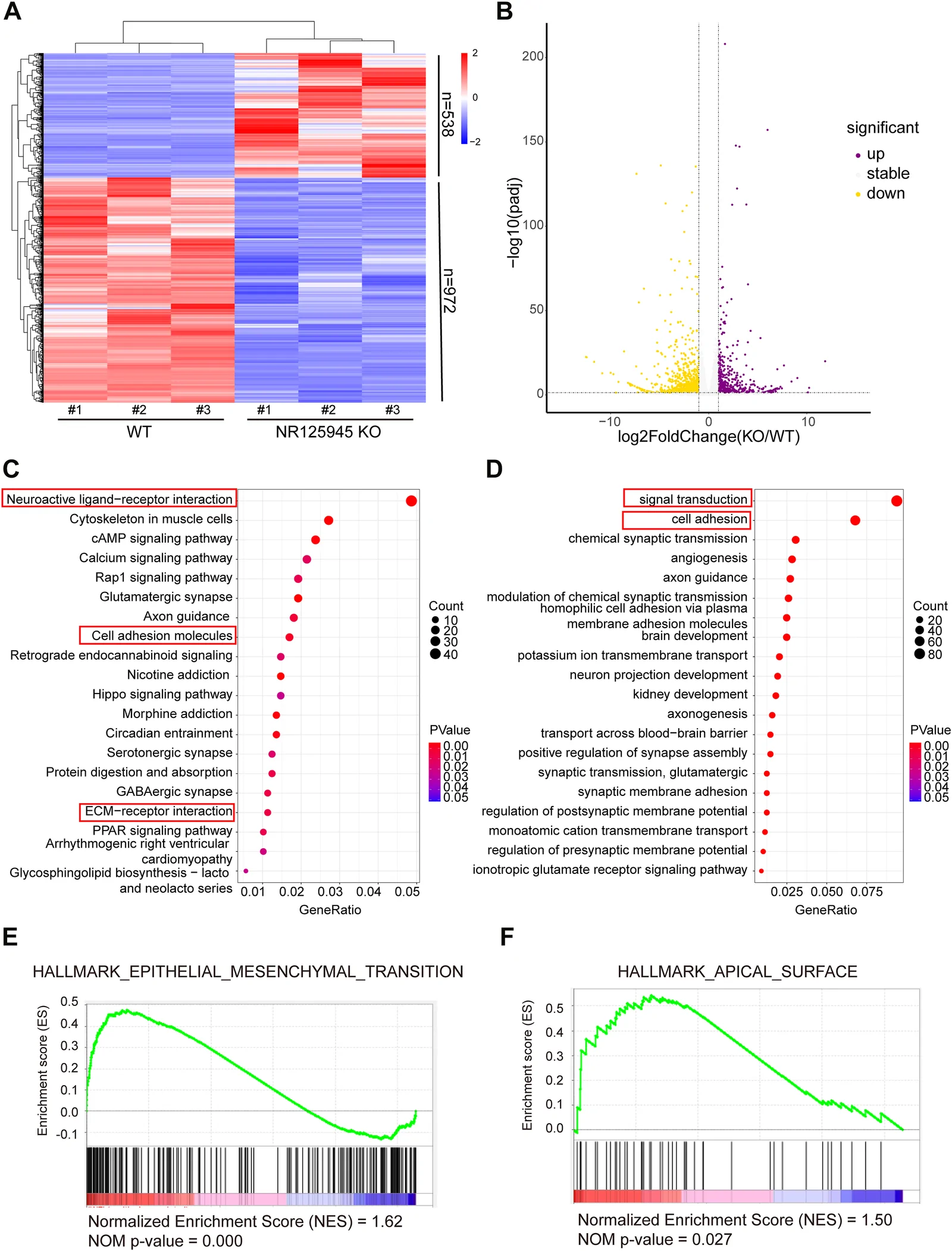
**Transcriptomic consequences of NR125945-SE disruption in prostate cancer cells.***A*, heatmap showing differentially expressed genes (DEGs) identified by RNA-seq from three biological replicates of wild-type (WT) and NR125945-SE KO cells. Gene clusters reflect robust transcriptional alterations upon SE loss. *B*, Volcano plot illustrating global expression changes between WT and NR125945-SE-KO cells. Significantly dysregulated genes were defined as |log_2_ fold change| > 1 and adjusted *p* < 0.05. *C*, KEGG pathway enrichment analysis of significantly downregulated genes following NR125945-SE deletion, highlighting disrupted oncogenic and metabolic pathways. *D*, gene ontology (GO) enrichment analysis of downregulated genes, highlighting impaired biological processes associated with cell proliferation and migration. (*E*, *F*) Gene set enrichment analysis (GSEA) of DEGs demonstrating coordinated suppression of hallmark oncogenic programs and transcriptional networks upon loss of the NR125945-SE.

### 4C mapping identifies *CAPN2*, *KRT19*, and *LAMB3* as candidate super-enhancer-regulated target genes

Since the super-enhancer is located in a gene desert region, we performed circular chromosome conformation capture sequencing (4C-seq) analysis utilizing a single bait fragment spanning chr1: 235,099,921–235,100,336 (hg19), corresponding to the core H3K27ac-enriched region of NR125945-SE, as “bait” to identify candidate target genes (Fig. S2, *A*–*C*). The 4C-seq analysis revealed that NR125945-SE directly interacts with 388 loci in cis and 92 loci in trans (Fig. 4*A*). We integrated 4C-seq and RNA-seq analysis to identify candidate transcriptional targets of NR125945-SE, ultimately identifying four genes, *CAPN2*, *LAMB3*, *KRT19*, and the non-coding transcript *LOC101927851* as potential targets positively regulated by NR125945-SE (Figs. 4*B* and S3, *A*–*C*). Surprisingly, with the exception of the NR125945-SE-derived transcript *LOC101927851*, all the three candidate target genes located at a distance from NR125945-SE in the genome. Both *CAPN2* and *LAMB3* are located on chr1, approximately 10 Mb and 25 Mb away from NR125945-SE, respectively, while *KRT19* is positioned on chr17. The four candidate genes were significantly downregulated upon NR125945-SE KO (Fig. 4*C*). Additionally, chromosome conformation capture (3C) assays provided supporting evidence for direct chromatin interactions between NR125945-SE and *CAPN2*, *LAMB3*, or *KRT19* (Fig. 4, *D*–*G*). Given that *LOC101927851* is a long non-coding RNA transcribed directly from the enhancer locus and exhibits extremely low expression levels, we focused our subsequent analyses primarily on the remaining three genes. These results indicated a link between the super-enhancer and three target genes, suggesting that the NR125945-SE forms long-range interactions with multiple targets which may form the regulatory network underlying PCa progression.

**Figure 4:**
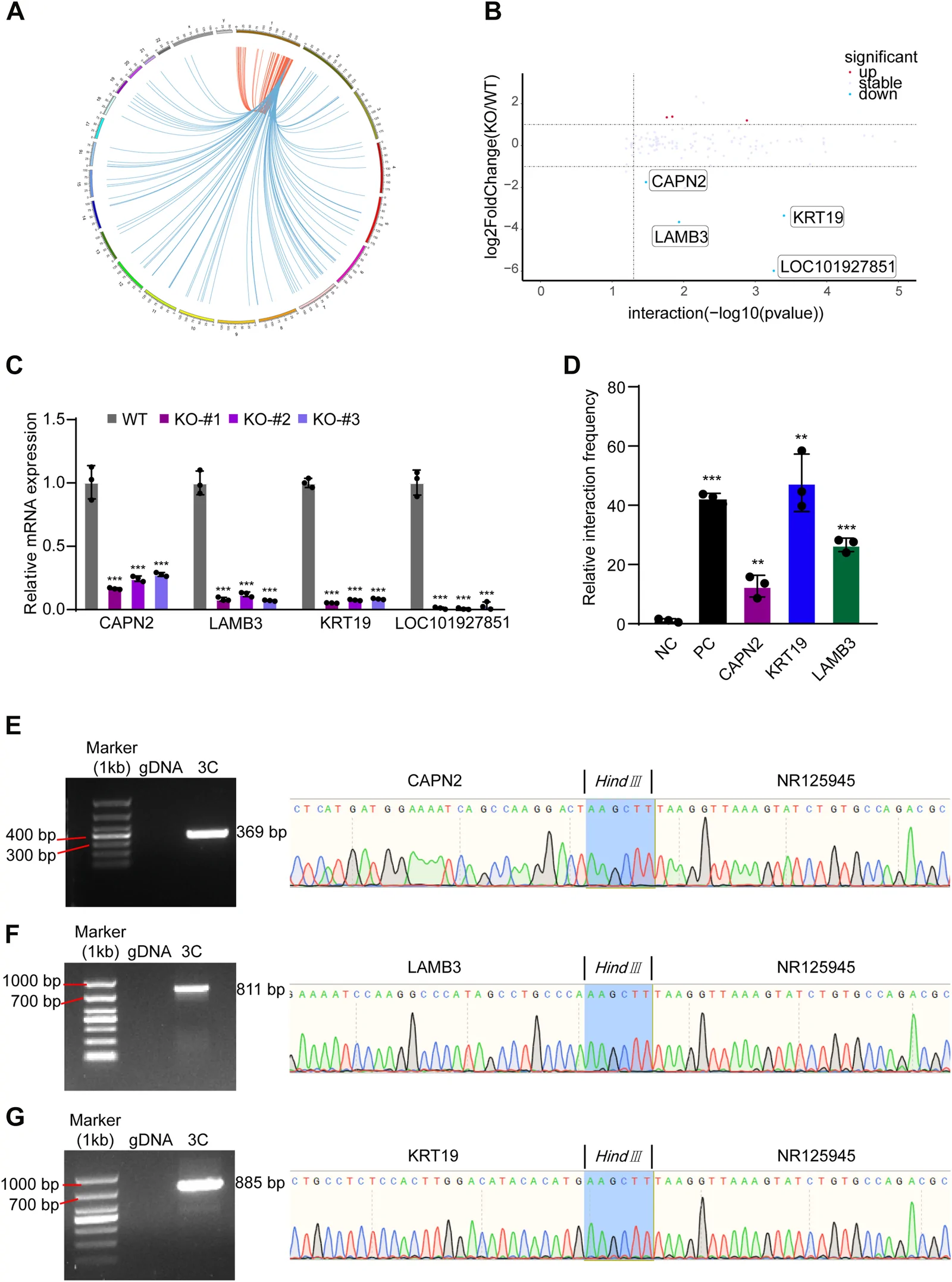
**Identification and validation of NR125945-SE–mediated long-range chromatin interactions and downstream effector genes.***A*, circos plot showing 4C-seq interaction profiles anchored at the NR125945-SE locus, revealing multiple distal chromatin regions engaged in long-range contacts. *B*, integration of RNA-seq and 4C-seq datasets to prioritize candidate target genes. The x-axis represents interaction significance from 4C-seq (–log_10_*p* value), and the y-axis represents transcriptional changes from RNA-seq (log_2_ fold change). Horizontal dashed lines indicate log_2_FC = 1 and −1; the vertical dashed line denotes –log_10_ (P) = 1.3. *C*, qPCR validation of candidate NR125945-SE-regulated genes. Data represent mean ± SD. ∗∗∗*p* < 0.001; ∗∗*p* < 0.01 (two-tailed unpaired Student's *t* test). *D*, 3C-qPCR validation of the interaction between the NR125945-SE and its candidate target genes. Data are presented as mean ± SD from three independent experiments. ∗∗∗*p* < 0.001; ∗∗*p* < 0.01 (two-tailed unpaired Student's *t* test). *E–G*, independent 3C assays confirming physical interactions between NR125945-SE and three candidate target genes: *CAPN2* (*E*), *LAMB3* (*F*), and *KRT19* (*G*). PCR products amplified from genomic DNA (gDNA) and 3C libraries are shown alongside DNA markers. Interaction-specific PCR primers were designed based on enhancer and gene-proximal restriction fragments.

### Rescue of *CAPN2* and *KRT19* restores phenotypes in NR125945-SE KO cells

Given that NR125945-SE forms long-range chromatin interactions with the three candidate genes *CAPN2*, *LAMB3*, and *KRT19*, we assessed their phenotypic contributions by re-expressing these candidate target genes in the super-enhancer knockout background in 22Rv1 cells (Figs. 5, *A*–*D* and S1, *A*, *B*). Upon re-expression in super-enhancer KO cells, *CAPN2* and *KRT19* over-expression cell lines showed partial rescue. Transwell and soft agar assays demonstrated that re-expression of either *CAPN2* or *KRT19* significantly rescued defects in invasive migration and decreased colony formation capacity caused by super-enhancer deletion (Fig. 5, *E*–*H*). However, *LAMB3* OE cell line exhibited clonal heterogeneity and correlated with incomplete phenotypic rescue (Fig. S4, *C* and *D*). Furthermore, we generated candidate genes CRISPRi cell lines (Fig. S4, *E*–*J*). The results showed that individual knockdown of *CAPN2* or *KRT19* significantly reduced cell migration in Transwell assays, as opposed to *LAMB3* KD, which had no significant effect on migration (Fig. 5, *I* and *J*). Notably, in public clinical-transcriptomic cohorts, higher expression of the NR125945-SE-regulated genes *CAPN2* and *KRT19* was significantly associated with poor biochemical recurrence–free survival (BCR-free survival) in patients with prostate cancer (Fig. S4, *K* and *L*). Taken together, our data support the conclusion that *CAPN2* and *KRT19* serve as downstream targets of the super-enhancer NR125945-SE in modulating PCa cell migration.

**Figure 5:**
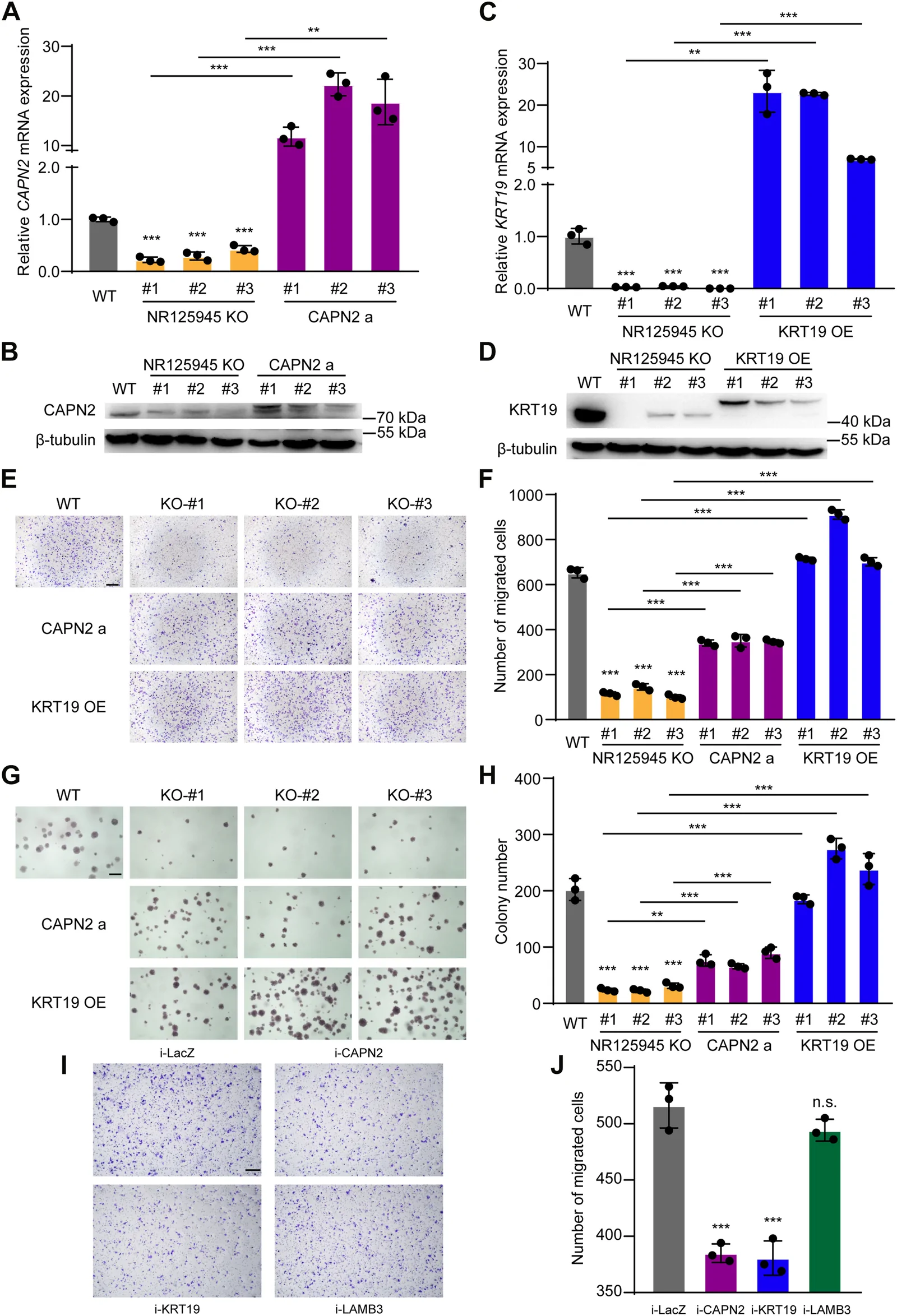
**Functional rescue of NR125945-SE downstream effector genes.***A and B*, rescue of *CAPN2* expression in NR125945-SE-knockout 22Rv1 cells after CRISPRa-mediated reactivation, assessed by qPCR (*A*) and representative Western blot (*B*). Data are presented as mean ± SD from three independent experiments. *C* and *D*, rescue of *KRT19* expression in NR125945-SE-knockout 22Rv1 cells after overexpression-mediated reactivation, assessed by qPCR (*C*) and representative Western blot (*D*). *E* and *F*, Transwell migration assays comparing WT 22Rv1 cells, three independent NR125945-SE knockout clones (KO1–KO3), and *CAPN2*- or *KRT19*-rescued KO cells. Migrated cells were stained with 0.1% crystal violet, with quantification shown in (*F*). Data are presented as mean ± SD from three independent experiments. *G* and *H*, soft agar colony formation assays performed in WT, NR125945-SE KO1–KO3, and *CAPN2*- or *KRT19*-rescued KO cells, with quantification shown in (*H*). *I* and *J*, Transwell assays assessing cell migration following individual knockdown of *CAPN2*, *KRT19*, or *LAMB3* (*I*). Migrated cells were stained with 0.1% crystal violet, with quantification shown in (*J*). All data is presented as mean ± SD from three independent experiments. ∗∗*p* < 0.01, ∗∗∗*p* < 0.001; n.s., not significant (two-tailed unpaired Student's *t* test).

To directly test whether these target genes functionally mediate the downstream transcriptional program of the super-enhancer, we performed individual re-expression of *KRT19* or *CAPN2* in SE-KO cells. Consistent with our experimental observations that re-expressing these genes could partially rescue the defects in cell migration caused by super-enhancer deletion, RNA-seq analysis revealed that *KRT19* or *CAPN2* significantly restored a substantial fraction of the transcriptomic signature disrupted in SE-KO cells (Fig. S5, *A*–*D*). Gene ontology (GO) analysis of the rescued genes provided molecular insights into the observed phenotypic recovery. Both target genes commonly rescued pathways involved in homophilic cell-cell adhesion and microvillus organization, while *KRT19* specifically regulated extracellular matrix (ECM) and structure organization (Fig. S5, *B* and *D*). These structural and adhesive changes are critical for cellular motility and invasion. These results indicate that *KRT19* and *CAPN2* are not merely passive marker genes but functionally contribute to the broader transcriptional network downstream of this super-enhancer, likely through their roles in cytoskeletal remodeling and adhesion signaling rather than direct transcriptional regulation. Nevertheless, these two genes account for only a partial, albeit substantial, portion of the super-enhancer-dependent transcriptional program, and additional downstream effectors remain to be identified.

### Multiple transcription factors mediating super-enhancer NR125945-SE regulation of target genes

Since transcription factors (TFs) drive the function of the super-enhancer (32, 33), to elucidate upstream regulator linking the SE to its candidate targets, we first performed a bioinformatic prediction of TFs potentially binding to this super-enhancer region by JASPAR. The top five ranked TFs were selected for experimental validation (Fig. 6*A*). However, ZNF135 and ZNF384 were expressed at extremely low levels in 22Rv1 cells, and were therefore not pursued further. For the remaining three candidates, *ZNF460*, *ETV5* and *FOXK1*, we performed CUT&Tag to assess their occupancy at the NR125945 super-enhancer. Among them, *ETV5* and *FOXK1* showed clear enrichment at the NR125945 region, whereas *ZNF460* did not exhibit an obvious enrichment peak (Fig. 6*B*). We then constructed *ETV5* and *FOXK1* KD cell lines and examined the expression of *CAPN2* and *KRT19* in the corresponding knockdown cell lines (Fig. 6, *C*–*F*). Knockdown of *ETV5* or *FOXK1* led to reduced expression of both *CAPN2* and *KRT19*, consistent with a role for these two transcription factors in regulating the downstream target genes of this super-enhancer (Fig. 6, *G* and *H*). These results suggest that these TFs may work cooperatively to influence the regulation of target genes by NR125945-SE. Overall, these results suggest a candidate regulatory model in which the super-enhancer controls target gene expression through specific transcription factors, providing insight into the impact on PCa progression.

**Figure 6:**
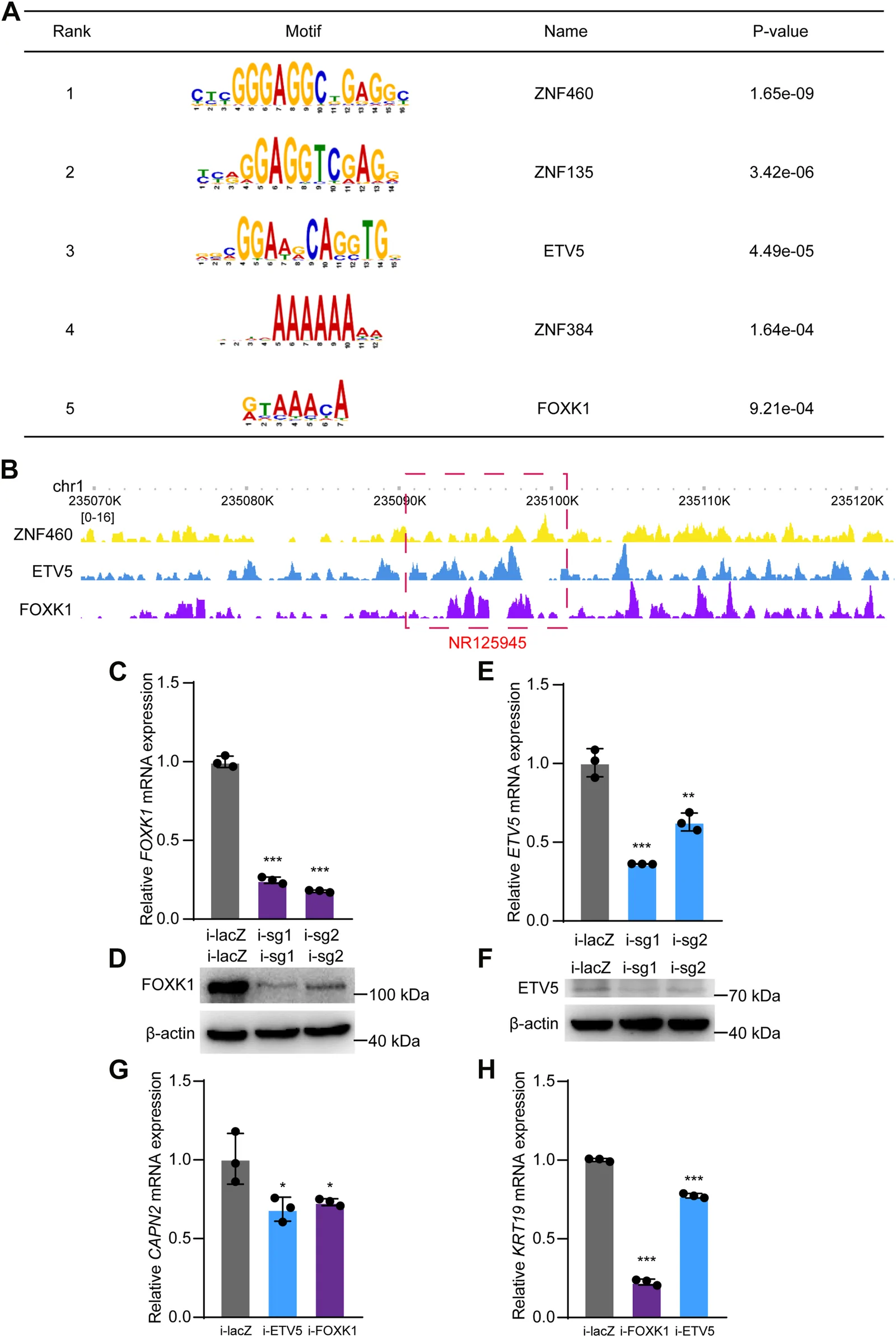
**Multiple transcription factors mediating super-enhancer NR125945-SE regulation of target genes.***A*, top five transcription factors predicted to bind the NR125945-SE regulatory region, ranked by motif enrichment *p* value. *B*, IGV visualization of TF binding (ZNF460, ETV5, and FOXK1) in the NR125945-SE region assessed by CUT&Tag. *C–D*, inhibition of *FOXK1* expression in 22Rv1 cells, assessed by qPCR (*C*) and representative Western blot (*D*). *E–F*, inhibition of *ETV5* expression in 22Rv1 cells, assessed by qPCR (*E*) and representative Western blot (*F*). *G-H*, qPCR validation of *CAPN2* (*G*) and *KRT19* (*H*) expression levels following CRISPRi-mediated knockdown of the identified transcription factors (ZNF460, ETV5, and FOXK1). Data are presented as mean ± SD from three independent experiments. ∗∗∗*p* < 0.001, ∗∗*p* < 0.01.

## Discussion

The functional annotation of genetic risk loci identified by GWAS and linkage analyses remains a central challenge in post-genomic cancer research. While the chromosomal interval 1q42.2-1q43 was identified as a hereditary prostate cancer susceptibility region over 2 decades ago, the specific regulatory elements and molecular mechanisms driving this association have remained largely elusive (34). In this study, we identified a tumor-specific super-enhancer, designated NR125945-SE, which is aberrantly hyperactivated in prostate cancer (PCa) cells compared to normal prostate epithelial cells. Notably, the lncRNA transcribed from the NR125945 locus is expressed at low levels across prostate cancer cell lines and clinical PRAD samples (Fig. S1, *B* and *C*). This observation supports a model in which the regulatory effects observed at this locus are primarily mediated by its super-enhancer activity at the chromatin level, rather than by the RNA transcript itself. Using CRISPR-mediated deletion combined with integrative multi-omics analysis, we show that NR125945-SE contributes to prostate cancer malignant progression and regulates the expression of *CAPN2* and *KRT19* through long-range chromatin interactions, identifying these genes as candidate downstream targets. Notably, this super-enhancer region is also epigenetically activated in human prostate cancer tissues and PDX samples (Fig. S1), extending the clinical relevance of our findings beyond cultured cell lines.

NR125945-SE resides within the 1q42.2–1q43 locus, which has been repeatedly associated with prostate cancer susceptibility in linkage and genetic studies. Accumulating evidence suggests that the vast majority of disease-associated genetic variants reside within non-coding regulatory elements, particularly super-enhancers (23, 35). Super-enhancers are highly sensitive to genetic perturbations; single-nucleotide polymorphisms (SNPs) within these regions can disrupt transcription factor binding motifs, thereby altering enhancer activity and dysregulating downstream target genes (36). By pinpointing NR125945-SE as a dominant regulatory hub within this susceptibility region, our findings align with the paradigm that "risk variants converge on super-enhancers." However, we emphasize that our current data do not directly test whether germline susceptibility variants modulate the activity of NR125945-SE, as we have not performed eQTL analysis, allele-specific epigenomic profiling, or variant-level perturbation. Future studies combining these approaches will be required to establish whether individual risk variants at this locus functionally influence NR125945-SE activity and thereby contribute to inherited prostate cancer susceptibility.

A key advance of our study is the elucidation of the long-range regulatory network driven by NR125945-SE. Through integrated RNA-seq and 4C-seq analysis, corroborated by knockout, re-expression, and rescue experiments, we identified *CAPN2* and *KRT19* as candidate downstream targets of this super-enhancer. CAPN2 (Calpain-2) is a calcium-dependent protease implicated in cytoskeletal remodeling and focal adhesion turnover, processes essential for cancer cell migration and invasion (37, 38). Similarly, KRT19 (Keratin 19), although typically an epithelial marker, has been linked to aggressive phenotypes, stem-like features, and poor prognosis in advanced PCa (39). Importantly, our rescue RNA-seq analysis showed that combined re-expression of these two genes in the super-enhancer knockout background partially rescued a substantial fraction of the dysregulated transcriptome, with the rescued genes enriched in cell adhesion, extracellular matrix organization, and cell migration pathways. These results indicate that *CAPN2* and *KRT19* are not merely passive marker genes but functionally contribute to the broader transcriptional network downstream of this super-enhancer, likely through their roles in cytoskeletal remodeling and adhesion signaling rather than through direct transcriptional regulation; however, these genes account for only a partial, albeit substantial, portion of the super-enhancer-dependent transcriptional program, and additional downstream effectors remain to be identified. The co-regulation of these genes suggests that NR125945-SE does not merely activate isolated genes but coordinates a specific transcriptional program governing cytoskeletal dynamics and invasive potential. These findings underscore the ability of super-enhancers to act as “molecular switches” that synchronize complex biological behaviors during tumorigenesis (40).

Furthermore, our analysis of the upstream regulatory network suggests a candidate model in which NR125945-SE activity is sustained by the cooperative binding of multiple transcription factors. Through motif prediction and CUT&Tag assays, we demonstrated that *ETV5* and *FOXK1* physically occupy the core region of this super-enhancer in 22Rv1 cells. These observations are consistent with a model in which these transcription factors contribute to the establishment or maintenance of the active enhancer chromatin state, and are reminiscent of the "phase separation" model, where high densities of transcription factors and co-activators form condensates to drive robust transcription (41). In prostate cancer, lineage-specific factors such as FOXA1, HOXB13, and AR often collaborate to reprogram the enhancer landscape (42, 43, 44, 45, 46). Our results imply that NR125945-SE serves as a convergence point for these oncogenic signaling pathways, ensuring the sustained high-level expression of target genes required for tumor maintenance. However, we acknowledge that our data do not yet establish whether the effects of these transcription factors are mediated directly at the super-enhancer or partly through independent mechanisms, and further dissection of their cooperative regulation will be required.

From a therapeutic perspective, the tumor-specificity of NR125945-SE highlights a regulatory feature that may be a promising avenue for treatment. Previous studies have shown that the inhibition of transcriptional co-activator associated with super-enhancer function, including BET inhibitors such as JQ1 and OTX015 as well as CDK7 inhibitor THZ1, can suppress super-enhancer-driven transcription across various cancer types (47, 48, 49, 50, 51). Our study identifies NR125945-SE as a functionally essential element, providing a rationale for pharmacological strategies aimed at disrupting this specific SE complex or its associated cofactors (52). Moreover, our clinical analyses showed that higher expression of *CAPN2* and *KRT19* was significantly associated with poor biochemical recurrence-free survival in prostate cancer patients (Fig. S4, *K* and *L*), suggesting that this super-enhancer–target axis is associated with advanced disease. Thus, expression of *CAPN2* and *KRT19* may be explored as prognostic indicators for characterizing high-risk patients, and the NR125945-SE complex may represent a therapeutic vulnerability in advanced prostate cancer.

Several limitations of this work should be acknowledged. First, most of our functional experiments were performed in a single prostate cancer cell line, 22Rv1. We attempted to generate super-enhancer knockout clones in C4-2B and LNCaP cells, but stable edited clones could not be obtained, most likely because these lines are highly polyploid, which complicates precise CRISPR-mediated large genomic deletion. Nevertheless, 4C-seq in C4-2B cells recapitulated key interacting candidate loci observed in 22Rv1 (Table S7), and public H3K27ac ChIP-seq data confirmed activation of this super-enhancer in human prostate cancer tissues (Fig. S1), supporting that this regulatory element is active beyond a single cellular model. In addition, this regulatory axis requires validation across diverse PCa subtypes and disease stages (53, 54). Second, the long-range, including inter-chromosomal, interactions between NR125945-SE and its candidate target genes were detected by 4C-seq but could not be independently confirmed by quantitative 3C-qPCR in our hands despite multiple attempts. We therefore emphasize that the functional relationship between this super-enhancer and these genes is supported by the concordant knockout, re-expression, and rescue data, whereas the underlying long-range chromatin contacts await further orthogonal validation. Third, although 4C-seq provides bait-centered, genome-wide interaction profiles, its results may not fully overlap with genome-wide multi-way methods such as Hi-C, HiChIP, or ChIA-PET, which recover fewer bait-distal and lower-frequency contacts. The limited overlap with public datasets is therefore not unexpected and is discussed above. Fourth, our study does not directly test whether germline susceptibility variants at the 1q42.2–1q43 locus affect NR125945-SE activity; eQTL analysis, allele-specific epigenomic profiling, and risk-variant perturbation will be required to establish such a link. Finally, expanding H3K27ac ChIP-seq analyses to larger clinical cohorts (55) incorporating single-cell and spatial transcriptomic analyses to clarify the heterogeneity and context-specificity of super-enhancer function within the tumor microenvironment (56, 57, 58) and employing higher-resolution chromatin conformation techniques, such as Hi-C or HiChIP, could further elucidate the dynamic nature of super-enhancer-mediated transcriptional regulation (59).

In conclusion, our work uncovers a prostate cancer-specific super-enhancer, NR125945-SE, within the 1q42.2-1q43 susceptibility region. By linking chromatin architecture to transcriptional regulation and malignant phenotypes, we provide mechanistic insight into how this super-enhancer contributes to PCa progression through its candidate downstream targets *CAPN2* and *KRT19*. We anticipate that future studies directly testing the interplay between genetic susceptibility variants and super-enhancer activity will further clarify the contribution of this locus to inherited prostate cancer risk. This study not only deepens our understanding of epigenetic dysregulation in prostate cancer but also highlights the potential for developing precision therapeutics that target oncogenic super-enhancers.

## Experimental procedures

### Cell culture and transfection

The human PCa cell line 22Rv1 cells were obtained from the American Type Culture Collection (ATCC). 22Rv1 cells were incubated at 37 °C with 5% CO_2_ and cultured in complete RPMI 1640 Medium modified (HyClone), supplemented with 10% fetal bovine serum (FBS) (Biological Industries) and 1% penicillin/streptomycin (Gibco). HEK-293T cells were obtained from the ATCC, incubated at 37 °C with 5% CO_2_ and cultured with 10% (v/v) FBS and 90% Dulbecco’s Modified Eagle Medium supplemented with 1% penicillin/streptomycin (Gibco). Cells were all *mycoplasma* free.

### CRISPR/Cas9-mediated deletions

To delete the entire super-enhancer NR125945-SE region, 22Rv1 cells were transfected with plasmids containing Cas9 and guide RNAs targeting that region. Colonies were derived from single cells and tested for target region deletion. Cell clones were genotyped, and three clones with homozygous deletion of the super-enhancer NR125945-SE region plus three control clones were used for RNA-seq analysis. Sequences of single guide RNAs and enhancer KO identified primers are listed in Table S1. SgRNAs were designed using a CRISPR design tool (https://zlab.bio/guide-design-resources).

### Lentivirus production

HEK-293T cells were seeded into a 10 cm plate and incubated overnight at 37 °C and 5% CO_2_. Cells were then transfected with 8 μg of targeting plasmid and 2 μg of pMD2.G (Addgene, 12259), and 5 μg of psPAX2 (Addgene, 12260) packaging plasmids using 24 μl of LipoFilterTM (HANBIO, HB-TRLF-1000). After transfection for 48 h, viral supernatants were collected and centrifuged at 3000×*g* for 10 min at 4 °C to remove cell debris. The supernatants were then aliquoted and stored at −80 °C.

### CRISPRi assay

A stable 22Rv1 CRISPRi cell line was generated using the Lenti-dCas9-KRAB-blast plasmid (89567, Addgene). In brief, lentiviral particles were generated in HEK-293T cells using the pMD2.G and psPAX2 packaging plasmids and then used to infect 22Rv1 cells for 6 to 8 h. Cells were then selected 5 to 7 days in 5 μg/ml blasticidin. CRISPRi sgRNAs sequences targeting genes regulated by the super-enhancer NR125945-SE were subsequently cloned into lentiGuide-Puro plasmid (52963, Addgene) using the restriction enzyme BsmBI. Lentiviral particles were generated, and stable CRISPRi cells were infected as described above and selected in 1 μg/ml puromycin for 2 to 3 days. RNA was then collected to assess gene expression levels. CRISPRi sgRNAs were designed using the GPP Web Portal (Broad institute; https://portals.broadinstitute.org/gpp/public/analysis-tools/sgrna-design). Sequences of single guide RNAs are shown in Table S1.

### CRISPRa assay

A stable CRISPRa cell line was generated using the lentiMPHv2 plasmid (89308, Addgene). Lentiviral particles were generated in HEK-293T cells using pMD2.G and psPAX2 packaging plasmids. Enhancer-KO cells were infected for 6 to 8 h and selected in 500 μg/ml hygromycin for 5 to 7 days. CRISPRa sgRNAs targeting *CAPN2* were designed and cloned into the lentiSAMv2 plasmid (75112, Addgene) using the restriction enzyme BsmBI, and packaged and infected as described above. Infected enhancer-KO CRISPRa cells were selected in 5 μg/ml blasticidin for 5 to 7 days and RNA was collected to assess gene expression levels. Note that only *CAPN2* was reactivated using the dCas9-based CRISPRa system; *KRT19* and *LAMB3* were overexpressed using lentiviral pLVX-based constitutive expression vectors or a PiggyBac (PB) transposon system, as described below. CRISPRa sgRNAs were designed using the GPP Web Portal (Broad institute; https://portals.broadinstitute.org/gpp/public/analysis-tools/sgrna-design). Sequences of single guide RNAs are provided in Table S1.

### Gene overexpression (lentiviral and PiggyBac)

For *KRT19* and *LAMB3* overexpression, coding sequences were cloned into lentiviral pLVX-based constitutive expression vectors or into a PiggyBac (PB) transposon vector. Lentiviral particles were generated in HEK-293T cells using pMD2.G and psPAX2 packaging plasmids, and stable lines were selected as described above. For the PB system, cells were co-transfected with the PB transposon plasmid and a transposase expression plasmid, and stable lines were selected with the puromycin.

### Migration

22Rv1 cells were trypsin-digested and resuspended in serum-free medium. 200 μl of the cell suspension (2 × 10^4^ cells per chamber) was seeded into the upper chambers of Transwell plates (8 μm pore size; Corning Costar), and 500 μl of complete RPMI 1640 Medium was added into the lower chambers. After a 24 h incubation at 37 °C, the inserts were gently washed with PBS, and the cells on the upper surface of the membranes were carefully removed with a cotton swab. The migrated cells were fixed with 4% fixative solution (Solarbio) and stained with 0.01% crystal violet. Cells were photographed and counted in three randomly selected fields using an inverted microscope.

### Colony formation

For the base layer, 1.5 ml of 1.2% agar (Lonza) diluted in DMEM medium was added per well in 6-well plates and incubated 30 min at 4 °C. Then, a top layer of 0.7% agar was mixed with 5 × 10^3^ cells and layered over the base layer, and plates were incubated again at 4 °C for 30 min. Cells were then cultured at 37 °C in a 5% CO_2_ incubator. After 14 to 20 days, images of colonies were taken, and quantification was performed.

### Public data acquisition and processing

Publicly available H3K27ac ChIP-seq data for prostate cancer cell lines and normal prostate tissues were obtained from the NCBI Gene Expression Omnibus (GEO) and ENCODE databases. The accession numbers are listed in Table S2. Raw sequencing reads were quality-controlled using FastQC and aligned to the human reference genome hg19 using Bowtie2 (60). In addition, public H3K27ac ChIP-seq datasets from localized prostate cancer (GSE130408), advanced prostate cancer (GSE289467), and NEPC PDX samples (GSE161948) were re-analyzed to assess H3K27ac signal at the NR125945-SE region in clinical specimens (Fig. S1). Publicly available H3K27ac HiChIP datasets in prostate cancer cells were also analyzed to examine interaction signals between the NR125945-SE region and the candidate target genes (Table S8).

### ChIP-seq analysis and super-enhancer identification

For ChIP-seq analysis, peaks were called using MACS2 with a q-value cutoff of 0.05. Differential binding analysis was performed using the DiffBind (http://bioconductor.org/packages/release/bioc/vignettes/DiffBind/inst/doc/DiffBind.pdf) (v3.8.4) R package. Differential peaks were identified based on an FDR < 0.05 and |log2 Fold Change| > 1. The significantly upregulated peaks in PCa cell lines are listed in Table S3. To identify super-enhancers (SEs), we utilized the ROSE (22, 40) (Rank Ordering of Super-Enhancers) algorithm.

### CUT&Tag assay

CUT&Tag assays were performed in 22Rv1 cells to profile the genome-wide occupancy of ZNF460, ETV5, and FOXK1, using antibodies against each transcription factor. Briefly, nuclei were isolated and incubated with antibody-conjugated protein A–Tn5 fusion protein, followed by tagmentation and PCR amplification of the released DNA fragments. Libraries were sequenced and aligned to the reference genome (hg19), and peaks were called using MACS2 with IgG control. Enrichment of each transcription factor at the NR125945-SE region was quantified, and representative IGV browser views are shown in Figure 6*B*. Peak calling statistics are summarized in Table S10.

### Chromosome conformation capture (3C) PCR assay

The 3C assay was performed in 22Rv1 cells as described (61). Briefly, 1 × 10^7^ cells were fixed in 1% formaldehyde and fractionated to obtain the nuclear fraction. Nuclear lysates were digested with 400 U HindIII overnight at 37 °C followed by addition of 4000 U T4 DNA ligase for *in situ* ligation at 16 °C overnight. DNA was extracted with phenol/chloroform and interactions between the super-enhancer NR125945-SE locus and the candidate partners were detected by PCR. Sequences of primers used for the analysis are provided in Table S1.

### Circular chromosome conformation capture (4C-seq) and analysis

The 4C assay was carried out using a published protocol (62) with some modifications. Briefly, cells were crosslinked with 1% formaldehyde to preserve chromatin conformations, followed by chromatin isolation and digestion with a primary restriction enzyme (DpnII). After intramolecular ligation under dilute conditions, a secondary restriction enzyme (CviQI) was used to linearise the chromatin loops, and the resulting fragments were self-ligated to form circular DNA templates. Inverse PCR was then carried out using viewpoint-specific primers to amplify the interacting fragments. 4C library was sequenced as paired-end reads using the Illumina Hiseq 4000 system.

For data analysis, raw reads were trimmed and aligned to the reference genome (hg19) using Bowtie2 (v2.4.4). To identify significant chromatin interactions at a genomic scale, we used the R/Bioconductor package r3Cseq (v1.30.0). Reads were assigned to restriction fragments across the entire genome using the “adjacentFragmentEndsReads” method. Interaction scores were normalized to RPM (reads per million). Significant interaction peaks were identified by comparing the observed frequency against a background power-law distribution model implemented in the getInteractions function, with a false discovery rate (FDR) < 0.05. Interaction profiles were visualized as circular plots and genome-track plots using the Integrative Genomics Viewer (IGV). The resulting interaction counts and peak annotations are provided in Table S4.

### RNA isolation and RT-qPCR

Total RNA was isolated from cultured cells using TRIzol reagent. 1 μg RNA was reverse-transcribed to cDNA using the PrimeScript RT Reagent Kit with a gDNA eraser Kit (Takara, no. RRO47B). RT-qPCR was performed with qPCR SYBR Green Master mix (YEASEN) using the CFX96 (Bio-rad). Relative target gene expression was analyzed using the comparative Ct method and normalized to expression levels of the housekeeping gene *HPRT1* or *GAPDH*. Sequences of RT-qPCR primers used for analysis are shown in Table S1.

### RNA-seq and analysis

Total RNA was isolated from cultured cells using TRIzol reagent. Total RNA was sent to Novogene for sequencing. Clean reads were aligned to the Ensembl hg38 human genome using Hisat2 (63) with default settings. Gene-level read counts were quantified using featureCounts (64). Differentially expressed genes (DEGs) between treatment and control groups were identified with DESeq2 (65), with significance thresholds set at adjusted *p* value < 0.05 and |log2 Fold Change| > 1. Gene ontology (GO) and KEGG analysis were performed using the DAVID online tool (66). The analysis results are shown in Table S5. For the rescue transcriptomic analysis, RNA-seq was performed in super-enhancer knockout cells re-expressing *CAPN2* and *KRT19* individually and differentially expressed genes relative to the knockout control were identified as described above; the complete results are provided in Table S9.

### Clinical cohort and public dataset analyses

Biochemical recurrence–free survival (BCR-free survival) analysis was performed using public prostate cancer patient clinical-transcriptomic cohorts. Patients were stratified by CAPN2 or KRT19 expression, and survival curves were compared using the log-rank test (Fig. S4, *K* and *L*). H3K27ac signals at the NR125945-SE region in clinical prostate cancer tissues and NEPC PDX samples were assessed by re-analyzing the GSE130408, GSE289467, and GSE161948 datasets.

### Western blot

Cells were lysed with RIPA buffer (Beyotime, P0013B) and the concentration of protein was measured by BCA Assay Kit (Beyotime, P0009). The samples were boiled with loading buffer in 95 °C for 5 min. Then samples were submitted to SDS-PAGE, transferred to PVDF membranes, and blocked by 5% BSA for 2 h. The membranes were incubated with primary antibodies at 4 °C overnight (CAPN2, Invitrogen, PA5-97534, 1:1000, KRT19, Abcam, ab76539, 1:1000, LAMB3, Proteintech, 26795-1-AP, 1:1000, ETV5, Abcam, ab102010, 1:1000, FOXK1, Abcam, ab18196, 1:1000, ZNF460, Proteintech, 25299-1-AP, 1:1000, β-TUBULIN, Beyotime, AF1216, 1:1000, β-ACTIN, SIMUBIOTECH, R9001M, 1:1000), followed by incubation with HRP-conjugated secondary antibodies for 2 h (Beyotime, A0208,1:2000). The membranes were wash by TBS for 5 min, 6 times. Then added the chemiluminescence detection buffer on the membrane and photoed by Bio-RAD ChemiDoc XRS^+^ system.

### Motif enrichment analysis

De novo motif discovery was performed on the genomic sequences underlying the NR125945-SE region using the MEME Suite (version 5.5.5) (67). Specifically, sequences in FASTA format were analyzed with the MEME tool under the following parameters: search for 5 motifs per run, motif width between 6 and 50 nucleotides, and an expected site distribution model of ‘Any Number of Repetitions’ (anr). The most significantly enriched motifs identified by MEME were then compared to known transcription factor binding profiles using the Tomtom tool (68), which queries the JASPAR 2022 CORE non-redundant vertebrate database. Matches were considered statistically significant with *p*-value of less than 0.05. The resulting motifs and their associated potential transcriptional regulators were visualized and summarized for downstream biological interpretation. The analysis results are shown in Table S6.

### Statistical analysis

All data was analyzed using Student’s *t* test. Statistically significant *p* values were indicated in figures as follows: ∗*p* < 0.05, ∗∗*p* < 0.01, ∗∗∗*p* < 0.001. N.S., not significant. Averages and SDs of at least three independent experiments are shown in figures, when applicable.

## Ethics approval and consent to participate

All methods were performed in accordance with the relevant guidelines and regulations. No animal and human research was conducted in this manuscript.

## Consent for publication

All authors read and approved the final manuscript.

## Data availability

The authors confirmed that the data supporting the findings of this study are available within the article (GSE346320, GSE346319, GSE346318). All additional data are available from corresponding authors upon request, please contact shijd@nankai.edu.cn.

## Supporting information

This article contains supporting information.

## Conflict of interest

The authors declare that they have no conflicts of interest with the contents of this article.
