# Supplementary material for: A prostate cancer–specific super-enhancer at 1q42.3 regulates the CAPN2 and KRT19
Source: J Biol Chem. 2026 Sep 23;302(11):113588. doi: 10.1016/j.jbc.2026.113588 (PMC13691572; doi:10.1016/j.jbc.2026.113588)
Supplement: Supplementary file 1 [file mmc1.docx]

**Supplemental information**

**A prostate cancer–specific super-enhancer at 1q42.3 regulates the *CAPN2* and *KRT19***

Zhenhao Zhao^1^, Xuan Liu^1^, Zeyun Yang^1^, Yuting Zhao^1^, Yuan Jiang^1^, Yunlong Bai^1^, Qin Wang^1^, Lei Zhang^1^, Zhongfang Zhao^1^, Yuyang Qian^2^, Hongxia Li^1^, Peng Li^1🖂^, Jiandang Shi^1🖂^, Wange Lu^1,3🖂^

Supplementary Figures

**Figure S1.** **The non-coding transcript NR125945.1 is consistently expressed at low levels across prostate cancer cell lines and clinical PRAD samples.**

(A) IGV visualization of published H3K27ac ChIP-seq tracks in the NR125945-SE region using prostate cancer tissues and patient-derived xenograft (PDX) models, including localized (GSE130408), advanced (GSE289467), and NEPC (GSE161948) samples.(B) The plot was generated from LncExpDB. The orange part represented the TPM of the non-coding transcript NR125945.1 across a panel of prostate cancer cell lines.(C) The clinical PRAD samples data was collected from TCGA and generated in GEPIA3. The red box represented PRAD tumor samples. The green box represented normal samples. Each dot represented individual samples. TPM, Transcripts Per Million.

**Figure S2.** **4C-seq analysis of the NR125945-SE bait region in 22Rv1 cells.**

(A) Schematic diagram of the 4C-seq procedure. The bait region (NR125945-SE, chr1:235099921-235100336) is indicated. The primary restriction enzyme used was DpnII, and the secondary enzyme was CviQI. (B) Genome-wide distribution of 4C-seq interacting loci across all chromosomes. Each red line represents a significant interaction identified with the bait region. (C) IGV visualization of the 4C-seq signal on chr1:235,014,529–235,178,307. The bait region (NR125945-SE) is marked by a red dashed box.

**Figure S3. IGV visualization of the 4C-seq signals of candidate target genes.**

(A-C) IGV visualization of the 4C-seq signals of CAPN2, KRT19 and LAMB3.

**Figure S4.** **Validation of NR125945-SE target genes and their clinical relevance.**

(A and B) Rescue of *LAMB3* expression in NR125945-SE-knockout 22Rv1 cells after overexpression-mediated reactivation, assessed by qPCR (A) and representative Western blot (B). Data are presented as mean ± SD from three independent experiments. ***P < 0.001; **P < 0.01 (two-tailed unpaired Student's *t*-test). (C and D) Transwell migration assays comparing WT 22Rv1 cells, three independent NR125945-SE knockout clones (KO1–KO3), and *LAMB3*-rescued KO cells. Migrated cells were stained with 0.1% crystal violet, with quantification shown in (D). Data are presented as mean ± SD from three independent experiments. ***P < 0.001; n.s., not significant (two-tailed unpaired Student's *t*-test). (E and F) Inhibition of *CAPN2* expression in 22Rv1 cells, assessed by qPCR (E) and representative Western blot (F). Data are presented as mean ± SD from three independent experiments. ***P < 0.001 (two-tailed unpaired Student's *t*-test). (G and H) Inhibition of *KRT19* expression in 22Rv1 cells, assessed by qPCR (G) and representative Western blot (H). Data are presented as mean ± SD from three independent experiments. ***P < 0.001 (two-tailed unpaired Student's *t*-test). (I and J) Inhibition of *LAMB3* expression in 22Rv1 cells, assessed by qPCR (I) and representative Western blot (J). Data are presented as mean ± SD from three independent experiments. ***P < 0.001 (two-tailed unpaired Student's *t*-test). (K and L) Kaplan–Meier survival analysis of prostate cancer patients stratified by high versus low expression of *CAPN2* (K) and *KRT19* (L) using publicly available datasets. HR, hazard ratio. Log-rank test was used for statistical analysis.

**Figure S5.** **Transcriptomic rescue analysis of *KRT19* and *CAPN2* in SE-KO cells.**

(A) Heatmap showing the expression profiles of top rescued genes in WT, NR125945-KO, and *KRT19* re-expressing cells (n = 3 per group). Selected genes (n = 50) were significantly down-regulated upon NR125945 knockout and significantly recovered following *KRT19* overexpression. Relative expression levels are represented by row-normalized Z-scores (red: high; blue: low). Samples are ordered by group, and genes are hierarchically clustered. (B) Dotplot of Gene Ontology (GO) enrichment analysis showing the top 15 biological process (BP) terms for genes rescued by *KRT19*. The x-axis represents the Gene Ratio, bubble size indicates the gene count, and the color gradient represents the Benjamini-Hochberg adjusted p-value. (C) Heatmap showing the expression profiles of top rescued genes in WT, NR125945-KO, and *CAPN2* re-expressing cells (n = 3 per group). Selected genes (n = 50) were significantly down-regulated upon NR125945 knockout and significantly recovered following *CAPN2* activation. Data visualization and scaling are performed as described in (A). (D) Dotplot of Gene Ontology (GO) enrichment analysis showing the top 15 biological process (BP) terms for genes rescued by *CAPN2*. Bubble size and color gradient are defined as described in (B).

**Figure S6.All of the uncropped Western blot images.**

**Supplementary Table legends**

Table S1. The sequences of designed CRISPR knockout sgRNA sequences, CRISPRa/i sgRNA sequences and the primers of 3C assays and qPCR assays.

Table S2. The public datasets used in the study.

Table S3. The upregulated peaks at 1q42.3-1q43 region in PCa cell lines.

Table S4. The results for 4C-seq analysis of NR125945-SE in 22Rv1 cell line.

Table S5. The results for RNA-seq analysis of WT and NR125945-SE KO.

Table S6. The results for motif discovery and enrichment analysis of NR125945-SE.

Table S7. The results for 4C-seq analysis of NR125945-SE in C4-2B cell line.

Table S8. Public H3K27ac HiChIP datasets analysis of interaction signals between the NR125945-SE region and the candidate target genes in prostate cancer cells.

Table S9. RNA-seq analysis of super-enhancer knockout cells re-expressing CAPN2 and KRT19 cell lines.

Table S10. CUT&Tag Peak calling statistics are summarized upon ZNF460, ETV5 and FOXK1.
